# Localized Electric Field Tailoring to Balance Voltage Reliability, Current Density, and High-Frequency Performance of AlGaN/GaN HEMTs

**DOI:** 10.3390/mi16111199

**Published:** 2025-10-22

**Authors:** Yuxin Wang, Jiangwen Wang, Zilong Dong, Peiran Tian, Yuxiu Liu, Junyi Zhai, Weiguo Hu

**Affiliations:** 1 Beijing Institute of Nanoenergy and Nanosystems, Chinese Academy of Sciences, Beijing 101400, China; wangyuxin@binn.cas.cn (Y.W.); wangjiangwen@binn.cas.cn (J.W.); dongzilong@binn.cas.cn (Z.D.); tianpeiran@binn.cas.cn (P.T.); liuyuxiu@binn.cas.cn (Y.L.); 2 School of Nanoscience and Engineering, University of Chinese Academy of Sciences, Beijing 100049, China

**Keywords:** AlGaN/GaN HEMT, breakdown voltage, cut-off frequency, technology computer aided design, non-uniform composite gate dielectric

## Abstract

Emerging applications including advanced industrial manufacturing, cutting-edge scientific research and medical equipment demand AlGaN/GaN HEMTs possessing both high-frequency and high-voltage characteristics. However, a persistent trade-off remains between the frequency characteristics and breakdown characteristics of these devices. In this study, we employed localized electric field tailoring (LEFT) by introducing materials with different dielectric constants to construct a non-uniform composite gate dielectric layer, aiming to balance the breakdown voltage and cut-off frequency of the device. Device models were developed using APSYS-2018 software and their reliability was experimentally validated. Research data indicates that, compared to traditional uniform high-k (typically with dielectric constants k > 10, such as HfO_2_ and HfZrO) gate dielectrics, the non-uniform composite gate dielectric structure demonstrates superior transconductance, saturation current density and cut-off frequency, with minimal degradation in breakdown voltage. Specifically, relative to HfO_2_ and HfZrO uniform devices, the Al_2_O_3_/HfO_2_ and Al_2_O_3_/HfZrO non-uniform HEMTs achieved 20.0% and 35.2% increases in cut-off frequency, respectively. Meanwhile, breakdown voltage remained above 97% of their uniform counterparts, saturation current density and transconductance increased by approximately 5%. Therefore, this non-uniform composite gate dielectric layer structure of AlGaN/GaN HEMT with LEFT holds great potential for industrial plasma generators, magnetic resonance imaging systems and biomedical radiofrequency hyperthermia devices.

## 1. Introduction

AlGaN/GaN heterojunction exhibits stronger spontaneous and piezoelectric polarization effects [1,2] compared to other III-V heterojunctions, generating a two-dimensional electron gas (2DEG) [3] at the interface. Consequently, AlGaN/GaN HEMTs demonstrate superior electrical characteristics, including high carrier concentration, mobility, and breakdown field [4,5,6], coupled with low on-resistance [7] and high switching speed [8]. Given the aforementioned advantages of AlGaN/GaN HEMTs and the inefficiency of Si-based devices at MHz frequencies, HEMTs integrating both high-frequency and high-voltage capabilities therefore demonstrate application prospects in emerging fields, including industrial plasma generators (e.g., PECVD devices), medical and scientific equipment (e.g., magnetic resonance imaging systems), and biomedical radiofrequency hyperthermia (e.g., 13.56 MHz tumor ablation systems).

However, the high-frequency performance and voltage withstand characteristics of AlGaN/GaN HEMTs are inherently trade-offs, posing significant challenges for co-optimization. Current breakdown voltage enhancement strategies include gate-drain spacing extension [9], gate lengthening [10], and MOS-structure integration [11]. Since dimensional scaling substantially increases on-resistance (R_on_), degrading power density and high-frequency characteristics, we implement MOS architectures for balanced performance. This approach deposits high-k gate dielectrics between metal and semiconductor interfaces [12], including hafnium-based oxides (e.g., HfO_2_ [13], HfSiO_x_ [14], HfZrO_x_ [15]), aluminum-based oxides (Al_2_O_3_ [16]), and zirconium-based oxides (ZrO_2_ [17]). In this context, “high-k” refers to dielectric materials with relative permittivity significantly higher than that of SiO_2_ (k ≈ 3.9), typically exceeding 10, such as HfO_2_ (k ≈ 25) and HfZrO (k ≈ 30). Al_2_O_3_ (k ≈ 9) is considered a moderate-to-high-k material. Although improving breakdown robustness, these high-k layers inevitably impose cut-off frequency penalties [18]. Notably, breakdown is typically localized, whereas frequency characteristics are uniform and holistic. This difference arises from non-uniform electric field distribution within the device: electric field concentration at the gate edge amplifies impact ionization, ultimately triggering localized avalanche breakdown. Conversely, the homogeneity of frequency characteristics relies on holistic carrier transport throughout the channel, which is governed by the statistically averaged properties of carriers (e.g., density, mobility, and velocity distribution) rather than local electric field peaks. Therefore, we propose localized reinforcement to enhance the device’s cut-off frequency while maintaining high breakdown voltage. Localized reinforcement refers to depositing the high-k dielectric layer at the drain-side gate edge where electric field crowding induces breakdown, while lower-k materials occupy low-field regions to minimize equivalent gate capacitance.

This study implements localized electric field tailoring (LEFT) through non-uniform composite gate dielectrics. High-k material at the gate-drain side enhances breakdown voltage, while lower-k material on the gate-source side improves cut-off frequency. This structure balances breakdown and frequency characteristics. Conventional uniform Al_2_O_3_ devices were fabricated for experimental validation, confirming model reliability. Additional devices incorporating HfO_2_ and HfZrO were systematically characterized. Compared to uniform high-k gate dielectrics, the proposed non-uniform composite structure effectively optimized electric field distribution while maintaining comparable breakdown voltage. More impressively, the cut-off frequency was significantly enhanced. These results demonstrate LEFT-enabled non-uniform gate dielectrics’ strong potential for practical applications.

## 2. Materials and Methods

**Figure 1:**
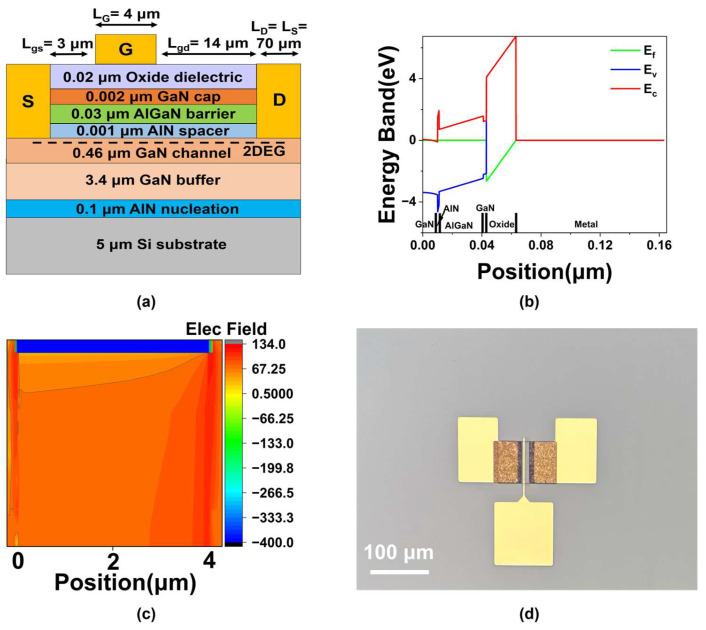
(**a**) The cross-section schematic of the AlGaN/GaN MOS-HEMT. (**b**) Band diagram of the MOS structure. (**c**) Two-dimensional electric field distribution under the gate in Al_2_O_3_ MOS-HEMT. (**d**) A micrograph of the Al_2_O_3_ MOS-HEMT.

Device models were developed using the APSYS platform, and corresponding devices were fabricated. Figure 1a illustrates the cross-sectional structure of the simulated device, with key geometric parameters provided in Table 1.

After device modeling, appropriate physical models and simulation parameters were applied to predict device behavior. Self-heating effects were simulated via an integrated thermal module, and breakdown characteristics were evaluated using an impact ionization model. The AlGaN/GaN heterojunction interface was treated with a polarization charge model. Besides the aforementioned physical models, the modified transferred-electron mobility model was also employed to accurately describe the current transport process. This model effectively hybridizes MTE and Canali models [19], as shown in Equations (1)–(3):

(1) $$v\left(E\right)={sin}^{2}\left(\frac{\pi }{2x}\right)\cdot v_{MTE}\left(E\right)+{cos}^{2}\left(\frac{\pi }{2x}\right)\cdot v_{C}\left(E\right),$$

(2) $$v_{MTE}\left(E\right)=\frac{F\left(E\right)+v_{sat}{\left(E/E_{MT}\right)}^{\beta_{T}}}{1+{\left(E/E_{MT}\right)}^{\beta_{T}}},$$

(3) $$v_{C}\left(E\right)=\frac{\mu_{low}E}{{\left(1+{\left(\mu_{low}E/v_{sat}\right)}^{\beta_{C}}\right)}^{1/\beta_{C}}},$$

where *V*(*E*) denotes the electron drift velocity, while *V_MTE_*(*E*) and *V_C_*(*E*) represent the electron drift velocities described by the MTE and Canali models, respectively. As shown, Equation (1) reduces to the MTE model when *x* = 1, and approaches the Canali model as *x* tends to 0. For the intermediate case of *x* = 0.5, Equation (1) incorporates an equal weighting of 50% from both the MTE and Canali models. The function *F*(*E*) is to imitate the kink in the low-field region, *V_sat_* denotes the saturation velocity, *E* represents the electric field, *E_MT_* is a fixed field value, *μ_low_* is the low-field mobility, and *β_T_* and *β_C_* correspond to the values of parameters beta_mte and beta_n in the model. 

Another important effect is the impurity dependence of the low field mobility, as shown in Equations (4) and (5):

(4) $$\mu_{0n}=\mu_{1n}+\frac{\left(\mu_{2n}-\mu_{1n}\right)}{1+{\left(\frac{N_{D}+N_{A}+\sum_{j}N_{tj}}{N_{rn}}\right)}^{\alpha_{n}}},$$

(5) $$\mu_{0p}=\mu_{1p}+\frac{\left(\mu_{2p}-\mu_{1p}\right)}{1+{\left(\frac{N_{D}+N_{A}+\sum_{j}N_{tj}}{N_{rp}}\right)}^{\alpha_{p}}},$$

where *μ*_0*n*_ and *μ*_0*p*_ are the low-field electron and hole mobilities, respectively; *μ*_1*n*_ and *μ*_1*p*_ are the carrier mobilities at high impurity concentration; *μ*_2*n*_ and *μ*_2*p*_ denote the carrier mobilities at low impurity concentration; *N_D_* and *N_A_* are the donor and acceptor concentrations; *Σ_j_N_tj_* is the total density of trap/defect levels summed over all considered trap types j; *N_rn_* and *N_rp_* are the empirical reference impurity concentrations; and *α_n_* and *α_p_* are empirical fitting exponents that determine how rapidly the mobility transitions with increasing total charged/neutral defect concentration for electrons and holes. 

A drift-diffusion model was used to govern the electrical behavior of the device. Fermi–Dirac statistics were employed to accurately calculate the carrier distribution of the high-concentration 2DEG, as shown in Equations (6)–(8):

(6) $$n=N_{c}F_{1/2}(\frac{E_{fn}-E_{c}}{kT}),$$

(7) $$p=N_{v}F_{1/2}(\frac{E_{v}-E_{fp}}{kT}),$$

(8) $$F_{1/2}(x)\approx {\left(e^{-x}+\xi (x)\right)}^{-1},$$

where *n* and *p* are the electron and hole concentrations; *N_C_* and *N_V_* are the effective density of states in the conduction and valence bands; *E_fn_* and *E_fp_* are the quasi-Fermi levels for electrons and holes; *E_C_* and *E_V_* are the conduction and valence band edges; *k* is the Boltzmann constant; *T* is the absolute temperature; *F*_1/2_ is the Fermi integral of order one-half; and *ξ*(*x*) is a correction function used in the approximate form of the Fermi integral. 

The Shockley–Read–Hall recombination model was utilized to simulate the carrier recombination process via defect levels in the material:

(9) $$R_{n}^{tj}=c_{nj}nN_{tj}\left(1-f_{tj}\right)-c_{nj}n_{1j}N_{tj}f_{tj},$$

(10) $$R_{p}^{tj}=c_{pj}pN_{tj}f_{tj}-c_{pj}p_{1j}N_{tj}(1-f_{tj}),$$

where $R_{n}^{tj}$ and $R_{p}^{tj}$ denote the electron and hole recombination rates via the *j*-th trap level; *c_nj_* and *c_pj_* are the capture coefficients for electrons and holes; *n* and *p* represent the free electron and hole concentrations; *n*_1*j*_ and *p*_1*j*_ are the carrier concentrations when the trap level equals the Fermi level; *N_tj_* is the trap density; and *J* is the trap occupancy probability.

The capture coefficients can be further expressed as follows:

(11) $$c_{nj}=\sigma_{nj}{\bar{v}}_{n}=\sigma_{nj}\sqrt{\frac{8kT}{\pi m_{n}}},$$

(12) $$c_{pj}=\sigma_{pj}{\bar{v}}_{p}=\sigma_{pj}\sqrt{\frac{8kT}{\pi m_{p}}},$$

where *σ_nj_* and *σ_pj_* are the capture cross-sections for electrons and holes; ${\bar{v}}_{n}$ and ${\bar{v}}_{p}$ are the average thermal velocities of electrons and holes; *m_n_* and *m_p_* represent the effective masses of electrons and holes. The trap or recombination center is completely specified by its density *N_tj_*, capture cross-sections *σ_nj_* and *σ_pj_*, and energy level *E_tj_*. The numerical values of the relevant physical parameters in Equations (1)–(12) are presented in Table 2. Figure 1b shows the energy band diagram of the MOS structure in Al_2_O_3_ MOS-HEMT. The conduction band of GaN on the left side contacts the Fermi level due to strong spontaneous and piezoelectric polarization effects. These effects lead to a high electron concentration in the channel layer, driving it into a degenerate state and forming a conductive channel. A two-dimensional electron gas is present at the interface between the Fermi level and the triangular quantum well. Figure 1c shows the two-dimensional electric field distribution under the gate in Al_2_O_3_ MOS-HEMT. The electric field is uniformly distributed, and the scale is calculated based on the actual electric field value of the formula 20log_10_.

To validate the simulation model, conventional Al_2_O_3_ gate dielectric HEMTs were fabricated. The process began with mesa isolation by ICP etching using a Cl_2_/BCl_3_/Ar mixture (10/25/5 sccm). Source and drain electrodes were formed via electron-beam deposition of Ti/Al/Ni/Au (20/120/45/55 nm), followed by rapid thermal annealing at 850 °C for 30 s in N_2_ to achieve ohmic contacts. A 20 nm Al_2_O_3_ gate dielectric was deposited by ALD at 160 °C, and Ni/Au (80/50 nm) was evaporated to form the Schottky gate. A micrograph of the completed Al_2_O_3_ MOS-HEMT is shown in Figure 1d.

## 3. Results

### 3.1. Matching Simulated and Measured Data for the MOS-HEMT

**Figure 2:**
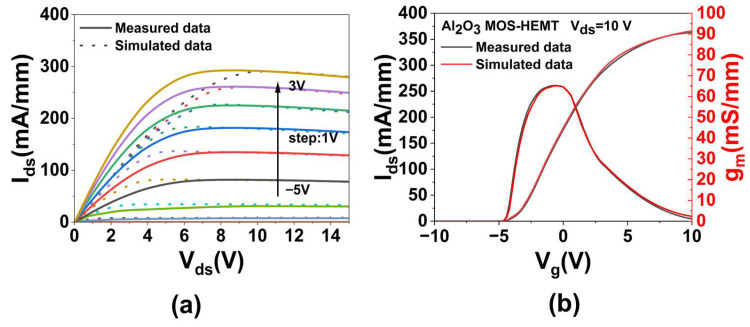
Measured and simulated results of the Al_2_O_3_ MOS-HEMT: (**a**) DC output performance, (**b**) transfer characteristics.

Following fabrication, the DC output performance and transfer characteristics of the devices were characterized using a semiconductor analyzer and compared with simulation data for accuracy validation. As shown in Figure 2a, the measured and simulated maximum drain current density (*I_ds,max_*) values were 292.6 and 290.6 mA/mm, respectively, representing a difference of 0.7%. Figure 2b presents the transfer characteristics, with maximum transconductance (*g_m,max_*) values of 65.32 and 65.12 mS/mm, and threshold voltage (*V_th_*) values of −4.80 and −4.77 V, showing a 0.3% and 0.6% discrepancy, respectively. To quantitatively verify the simulation accuracy, Table 3 compares the electrical characteristics extracted from the simulation and experiment, showing excellent consistency with deviations below 1%. These results demonstrate good agreement between measurement and simulation, confirming the reliability of the models.

### 3.2. Mechanism of Localized Electric Field Tailoring by Non-Uniform Composite Gate Dielectrics

**Figure 3:**
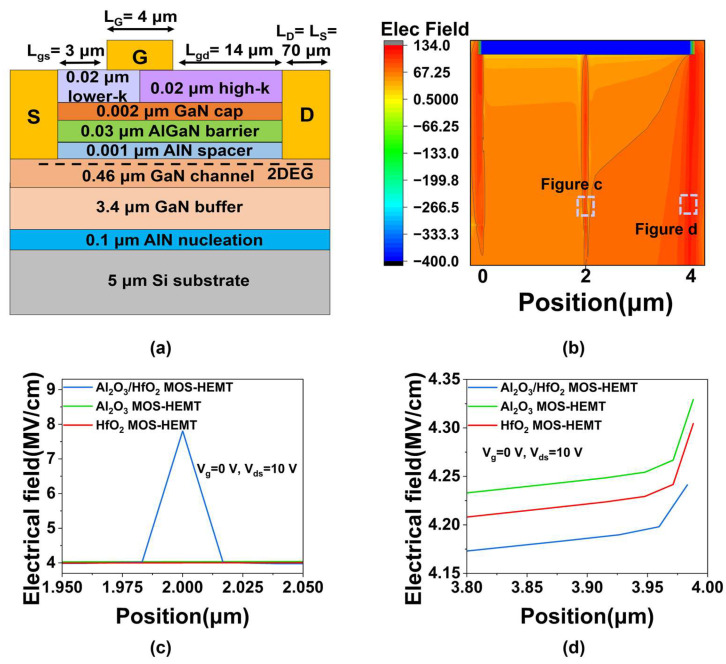
(**a**) The schematic of the non-uniform composite gate dielectric structure. (**b**) Two-dimensional electric field distribution under the gate in Al_2_O_3_/HfO_2_ MOS-HEMT. The transverse electric field distribution in the channel (**c**) directly under the gate dielectric layer, (**d**) at the drain-side gate edge of Al_2_O_3_, HfO_2_, and Al_2_O_3_/HfO_2_ MOS-HEMTs.

The schematic diagram of the non-uniform composite gate dielectric structure and the electric field distribution of Al_2_O_3_, HfO_2_, and Al_2_O_3_/HfO_2_ MOS-HEMTs are shown in Figure 3. Figure 3a presents the schematic of the non-uniform composite gate structure, incorporating high-k dielectrics (e.g., HfO_2_ and HfZrO) on the gate-drain side and a lower-k dielectric (e.g., Al_2_O_3_) on the gate-source side. Figure 3b displays the two-dimensional electric field distribution under the gate in the Al_2_O_3_/HfO_2_ MOS-HEMT, showing a peak at the 2 μm interface between the two dielectrics. Correspondingly, Figure 3c illustrates the transverse electric field profiles under the gate for Al_2_O_3_, HfO_2_, and Al_2_O_3_/HfO_2_ MOS-HEMTs. While the fields are uniform in the single-dielectric devices, a peak occurs at the dielectric interface in the composite structure, consistent with the observation in Figure 3b. This field discontinuity arises from the permittivity difference between HfO_2_ and Al_2_O_3_; the potential is continuous in the direction perpendicular to the interface, leading to a discontinuous jump in electric field strength. According to Gauss’s law, the electric fields perpendicular to the interface satisfy [20]

(13) $$\varepsilon_{1}E_{1}=\varepsilon_{2}E_{2},$$

where *ε*_1_ and *ε*_2_ denote the dielectric constants of HfO_2_ and Al_2_O_3_, respectively, and *E_1_* and *E*_2_ are the corresponding perpendicular electric fields. This field jump introduces a transverse electric field peak, enabling LEFT via regional control of dielectric constants. Figure 3d compares the transverse electric field at the drain-side gate edge for the three configurations, with values of 4.33, 4.30, and 4.24 MV/cm, respectively. The reduced field in the composite structure alleviates drain-edge field crowding and suppresses the hot electron effect, thereby enhancing reliability.

### 3.3. Analysis of Simulation Results

**Figure 4:**
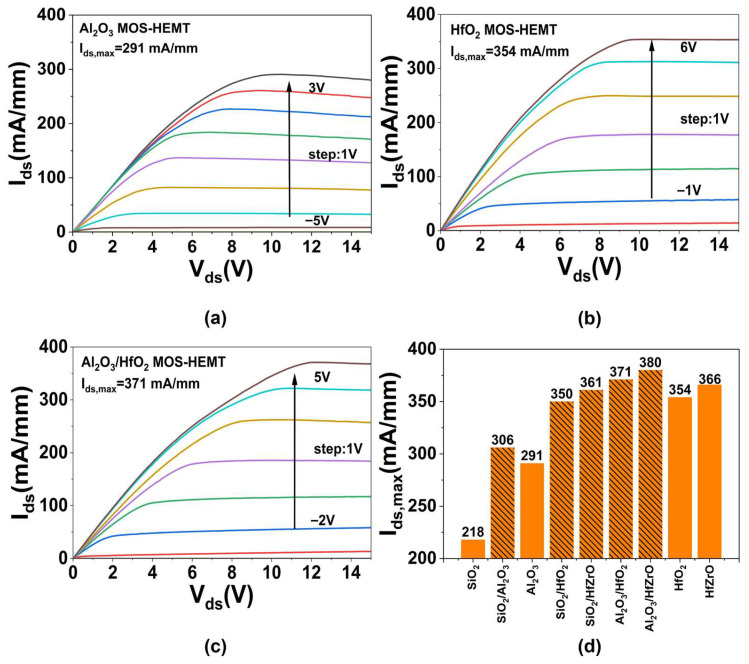
The DC output performances (*I_ds_*-*V_ds_*) of the (**a**) Al_2_O_3_, (**b**) HfO_2_, (**c**) Al_2_O_3_/HfO_2_ MOS-HEMT. (**d**) The *I_ds,max_* of all the devices.

The DC output characteristics of the MOS-HEMTs are shown in Figure 4. As shown in Figure 4a, the Al_2_O_3_ MOS-HEMT operates under a gate voltage ranging from −5 V to 3 V and achieves an *I_ds,max_* of 291 mA/mm. Beyond the saturation region, a discernible current reduction occurs, attributed to self-heating that elevates the heterojunction temperature and degrades output performance [21]. In contrast, the HfO_2_ MOS-HEMT (Figure 4b) is biased from −1 V to 6 V due to its higher *V_th_* and positive bias stability, delivering an *I_ds,max_* of 354 mA/mm without significant current decrease. This represents a 22% improvement over the Al_2_O_3_ device, resulting from the higher dielectric constant of HfO_2_, which reduces the depletion region width and source–drain resistance, thereby enhancing channel conductivity [22]. Moreover, analysis based on interface and trap models indicates that the increased permittivity also mitigates surface scattering and carrier trapping [23] at the metal/AlGaN junction, which in turn increases channel carrier concentration [24]. Figure 4c shows the Al_2_O_3_/HfO_2_ MOS-HEMT, gated from −2 V to 5 V with *V_th_* and positive bias stability intermediate between the single-dielectric devices. It exhibits an *I_ds,max_* of 371 mA/mm and minimal current degradation. The improved current stability in both HfO_2_ and Al_2_O_3_/HfO_2_ stems from the stronger electric field shielding under the gate, which reduces surface field intensity [25] and suppresses dynamic charge trapping/detrapping. The high-k dielectric also alleviates self-heating, enhancing reliability [26]. Finally, as summarized in Figure 4d, the simulated *I_ds,max_* of nine devices confirms that composite gate dielectrics achieve better performance compared to uniform higher-k layers, demonstrating that non-uniform composite gate dielectric devices with LEFT can deliver enhanced output characteristics.

**Figure 5:**
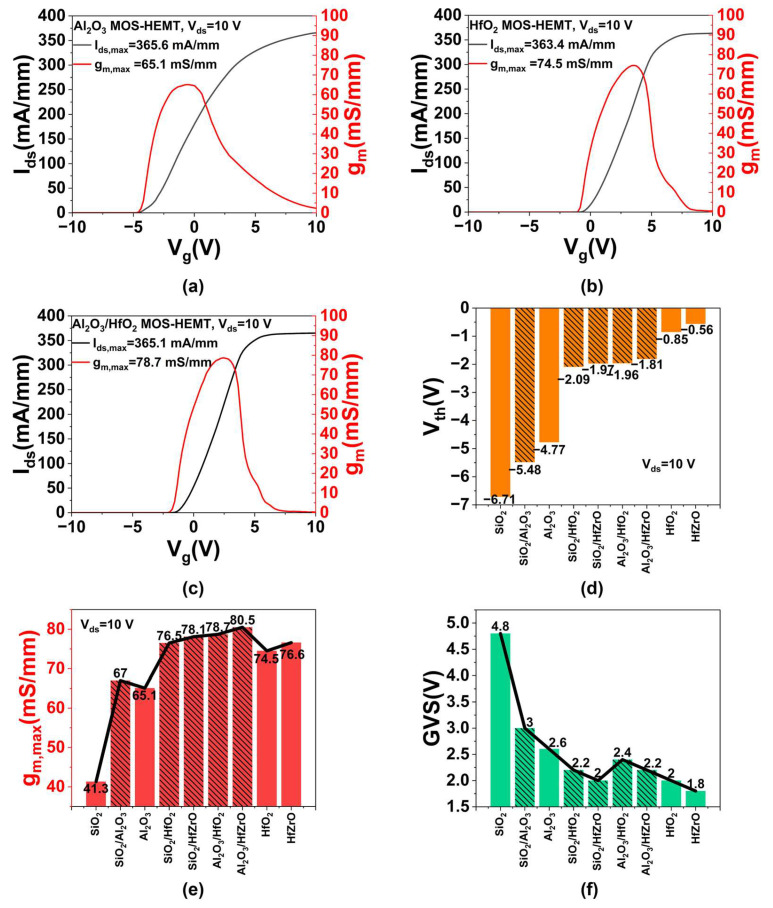
The transfer characteristics of (**a**) Al_2_O_3_, (**b**) HfO_2_ and (**c**) Al_2_O_3_/HfO_2_ MOS-HEMT. The (**d**) *V_th_*, (**e**) *g_m,max_* and (**f**) GVS values of all devices.

Figure 5a presents the transfer characteristics of the Al_2_O_3_ MOS-HEMT at *V_ds_* = 10 V and *V_g_* swept from −10 V to 10 V, yielding a *g_m,max_* of 65.1 mS/mm and a *V_th_* of −4.77 V. For the HfO_2_ MOS-HEMT (Figure 5b), *g_m,max_* reaches 74.5 mS/mm and *V_th_* is −0.85 V. The 14.4% improvement in *g_m,max_* is attributed to the higher dielectric constant, which enhances gate control capability [27,28]. The positive shift in *V_th_* also results from the increased permittivity [29]. As shown in Figure 5c, the Al_2_O_3_/HfO_2_ MOS-HEMT achieves a *g_m,max_* of 78.7 mS/mm and *V_th_* of −1.96 V. The further 5.6% increase in *g_m,max_* over the HfO_2_ MOS-HEMT arises from LEFT, which optimizes field distribution and improves electron transport efficiency. The negative *V_th_* shift relative to the HfO_2_ device is due to the reduced unit-area gate capacitance caused by the incorporation of the lower-k material. Based on the parallel-plate capacitance formula [30]:

(14) $$C=\frac{\varepsilon S}{4\pi kd},$$

where *C* represents the gate capacitance, *ε* denotes the absolute permittivity of dielectric layer (*ε = ε*_0_*ε_r_*), *S* is the gate area, *k* is the Coulomb’s constant, and *d* represents the thickness of the gate dielectric layer. The threshold voltage can be expressed as follows [31]:

(15) $$V_{T}=\frac{\sqrt{2\varepsilon_{s}qN_{A}\left(2\psi_{B}\right)}}{C_{0}}+2\psi_{B},$$

where *V_T_* represents the threshold voltage, *ε_s_* denotes the semiconductor permittivity, *q* is the elementary charge, *N_A_* is the acceptor concentration, *ψ_B_* is the bulk potential, and *C_0_* is the unit-area gate capacitance derived from Equation (14). The numerical values of the relevant physical parameters in Equations (14) and (15) are presented in Table 4. As the gate capacitance decreases, the absolute value of *V_th_* increases accordingly. Figure 5d,e summarize *V_th_* and *g_m,max_* across nine devices. The composite dielectric devices exhibit slightly higher *g_m,max_* than their uniform high-k counterparts, consistent with previous trends. Figure 5f compares the gate voltage swing (GVS), defined as the *V_g_* variation range where *g_m,max_* remains within 90% of its peak. GVS generally decreases with increasing dielectric constant, though composite dielectrics show improved GVS over uniform high-k layers. A higher GVS indicates enhanced linearity, which helps reduce phase noise, intermodulation distortion, and extends dynamic range in practical applications [32].

**Figure 6:**
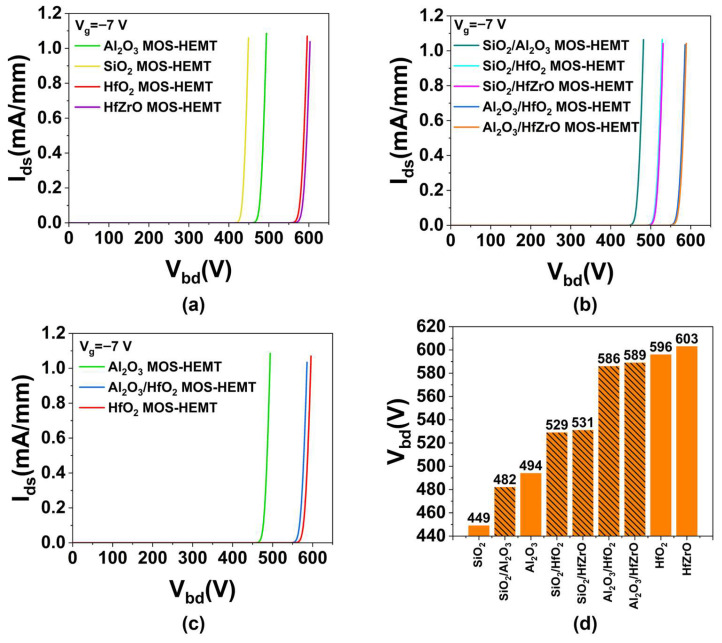
The *V_bd_* of MOS-HEMTs with (**a**) uniform gate dielectrics, (**b**) non-uniform composite gate dielectrics and (**c**) Al_2_O_3_, HfO_2_, Al_2_O_3_/HfO_2_ gate dielectrics. (**d**) The *V_bd_* values of all devices.

The breakdown voltage (*V_bd_*) is defined as the *V_ds_* at which *V_bd_* exceeds 1 mA/mm while *V_g_* is held at −7 V to maintain the off-state. Figure 6a shows the *V_bd_* of four uniform gate dielectric devices. With increasing dielectric constant, *V_bd_* rises significantly [33]. Similarly, Figure 6b presents *V_bd_* for five composite dielectric devices, where *V_bd_* also increases with equivalent permittivity. Figure 6c compares *V_bd_* among Al_2_O_3_, HfO_2_, and Al_2_O_3_/HfO_2_ MOS-HEMTs. The Al_2_O_3_/HfO_2_ MOS-HEMT exhibits *V_bd_* nearly identical to the HfO_2_ device, with only a 1.7% reduction, attributable to non-uniform field distribution leading to localized breakdown near the gate-drain side [34], rather than uniform breakdown. Figure 6d summarizes *V_bd_* across all nine devices. *V_bd_* increases consistently with dielectric constant from left to right. Moreover, the breakdown voltages of SiO_2_/Al_2_O_3_, Al_2_O_3_/HfO_2_, and Al_2_O_3_/HfZrO MOS-HEMTs closely match those of their uniform higher-k counterparts, confirming the feasibility and effectiveness of LEFT in improving breakdown performance and device reliability.

**Figure 7:**
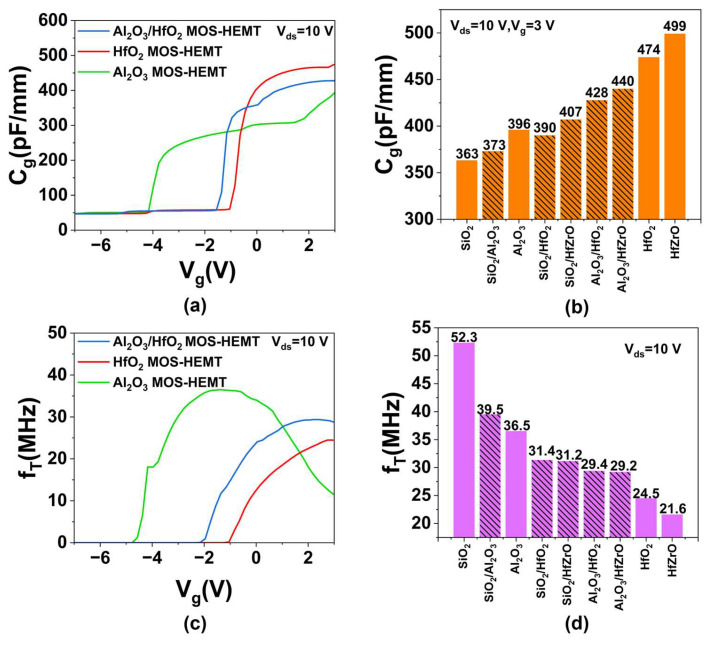
(**a**) The *C_g_*-*V_g_* characteristics of Al_2_O_3_, HfO_2_, and Al_2_O_3_/HfO_2_ MOS-HEMT. (**b**) The *C_g_* values of all devices. (**c**) The *f_T_*-*V_g_* characteristics of Al_2_O_3_, HfO_2_, and Al_2_O_3_/HfO_2_ MOS-HEMT. (**d**) The maximum *f_T_* values of all devices.

The device’s gate capacitance and cut-off frequency are shown in Figure 7. Figure 7a presents the *C_g_*-*V_g_* characteristics of Al_2_O_3_, HfO_2_, and Al_2_O_3_/HfO_2_ MOS-HEMTs at *V_ds_* = 10 V. As *V_g_* increases, *C_g_* rises slowly initially, increases rapidly in the linear region, and flattens out in the saturation region, reaching a maximum at *V_g_* = 3 V. The *C_g_* values are 396, 474, and 428 pF/mm, respectively, differing mainly due to the dielectric constants of the materials [35]. Figure 7b summarizes the simulated *C_g_* for nine devices, showing a positive correlation with dielectric constant. Figure 7c presents the *f_T_*-*V_g_* curves for the three devices. The Al_2_O_3_ MOS-HEMT peaks at *f_T_* = 36.5 MHz (*V_g_* = −1.4 V), while the HfO_2_ and Al_2_O_3_/HfO_2_ devices peak at 24.5 MHz (*V_g_* = 2.8 V) and 29.4 MHz (*V_g_* = 2.2 V), respectively. Based on the approximation [36]:

(16) $$f_{T}=\frac{g_{m}}{2\pi C_{g}}$$

the increase in *f_T_* is mainly due to reduced *C_g_*, with a minor contribution from increased g_m_. As shown in Figure 7d, *f_T_* generally decreases with higher dielectric constant [37]. Compared to their uniform higher-k counterparts, the Al_2_O_3_/HfZrO, SiO_2_/Al_2_O_3_, SiO_2_/HfO_2_, and SiO_2_/HfZrO MOS-HEMTs show *f_T_* improvements of 35.2%, 8.2%, 28.2%, and 44.4%, respectively. These results demonstrate that composite gate dielectrics with LEFT effectively alleviate the *f_T_* degradation typical of high-k devices, enabling balanced frequency performance.

## 4. Discussion

**Figure 8:**
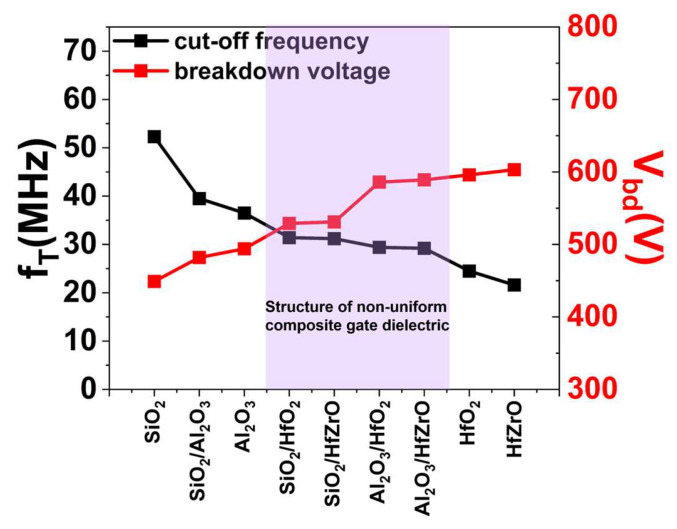
A summary of breakdown voltage and cut-off frequency of nine different dielectric passivation structures for AlGaN/GaN MOS-HEMT.

This study simulates and analyzes the electrical characteristics of various gate dielectric structures. The data indicates that the non-uniform composite gate dielectric device with LEFT exhibits *V_bd_* on par with that of uniform higher-k devices, while simultaneously delivering superior *g_m,max_*, *I_ds,max_* and *f_T_*. Specifically, compared to HfO_2_ and HfZrO MOS-HEMTs, the Al_2_O_3_/HfO_2_ and Al_2_O_3_/HfZrO structures show *f_T_* improvements of 20.0% and 35.2%, respectively, with only marginal *V_bd_* reductions of 1.7% and 2.3%, as summarized in Figure 8.

## 5. Conclusions

In summary, we propose a LEFT technique and develop corresponding device structures and TCAD models for both conventional uniform and non-uniform composite gate dielectrics. The simulation models were validated against experimental measurements. The LEFT technique retains a high-k dielectric on the gate-drain side while introducing a lower-k material on the gate-source side. This configuration modulates the electric field distribution, improves channel transport efficiency, and achieves an optimal trade-off between the two critical yet competing metrics: breakdown voltage and cut-off frequency. The core advantage of a non-uniform composite gate dielectric structure lies in its functional zoning. This provides a new approach to resolving the inherent contradictions in HEMTs. The AlGaN/GaN MOS-HEMT with a non-uniform composite gate dielectric structure, shows significant potential for high-voltage and high-frequency applications, such as industrial plasma generators, magnetic resonance imaging systems and biomedical radiofrequency hyperthermia devices.

## Figures and Tables

**Table 1:** Specific geometric parameters of the AlGaN/GaN MOS-HEMT.

Parameters	Value (µm)
*L_G_*	4
*L_S_*	70
*L_D_*	70
*L_gs_*	3
*L_gd_*	14
*W_G_*	100
*W_S_*	100
*W_D_*	100
Oxide dielectric layer	0.02
GaN cap layer	0.002
Al_0.3_Ga_0.7_N barrier layer	0.03
AlN spacer layer	0.001
GaN channel layer	0.46
Al_0.5_Ga_0.5_N buffer layer	3.4
AlN nucleation layer	0.1
Si substrate layer	5

**Table 2:** Relevant physical parameters in Equations (1)–(12).

Parameters	Units	Value
*V_sat_*	cm/s	1.91 × 10^7^
*E_MT_*	kV/cm	257
*μ_low_*	cm^2^/V·s	1000
*β_T_*	-	5.7
*β_C_*	-	1.7
*μ*_0*n*_	cm^2^/V·s	1200
*k*	eV/K	8.617 × 10^−5^
*T*	K	300
*N_tj_*	cm^−3^	1.85 × 10^16^
*σ_nj_*	cm^2^	1 × 10^−15^

**Table 3:** Comparison between simulated and measured characteristics.

Parameters	Measurement	Simulation	Error (%)
*I_ds,max_*	292.6 mA/mm	290.6 mA/mm	0.7
*g_m,max_*	65.32 mS/mm	65.12 mS/mm	0.3
*V_th_*	−4.80 V	−4.77 V	0.6

**Table 4:** Representative physical parameters in Equations (14) and (15).

Parameters	Units	Value
*ε_0_*	F/cm	8.85 × 10^−14^
*ε_r_(SiO*_2_ *)*	-	3.9
*ε_r_(Al*_2_ *O*_3_ *)*	-	9
*ε_r_(HfO*_2_ *)*	-	25
*ε_r_(HfZrO)*	-	30
*k*	N·m^2^/C^2^	8.99 × 10^9^
*d*	nm	20
*q*	C	1.602 × 10^−19^

## Data Availability

The raw data supporting the conclusions of this article will be made available by the authors on request.
